# Supplementary material for: Soil microbiota and herbivory drive the assembly of tomato plant-associated microbial communities through different mechanisms
Source: Commun Biol. 2024 May 13;7:564. doi: 10.1038/s42003-024-06259-6 (PMC11091179; doi:10.1038/s42003-024-06259-6)
Supplement: Supplementary file 2 — Supplementary material [file 42003_2024_6259_MOESM2_ESM.pdf]

Supplementary Material

Soil microbiota and herbivory drive the assembly of tomato plant-associated microbial communities through different mechanisms

Antonino Malacrinò, Alison E. Bennett

Contents

Supplementary figures 1

Supplementary tables 6

Supplementary figures

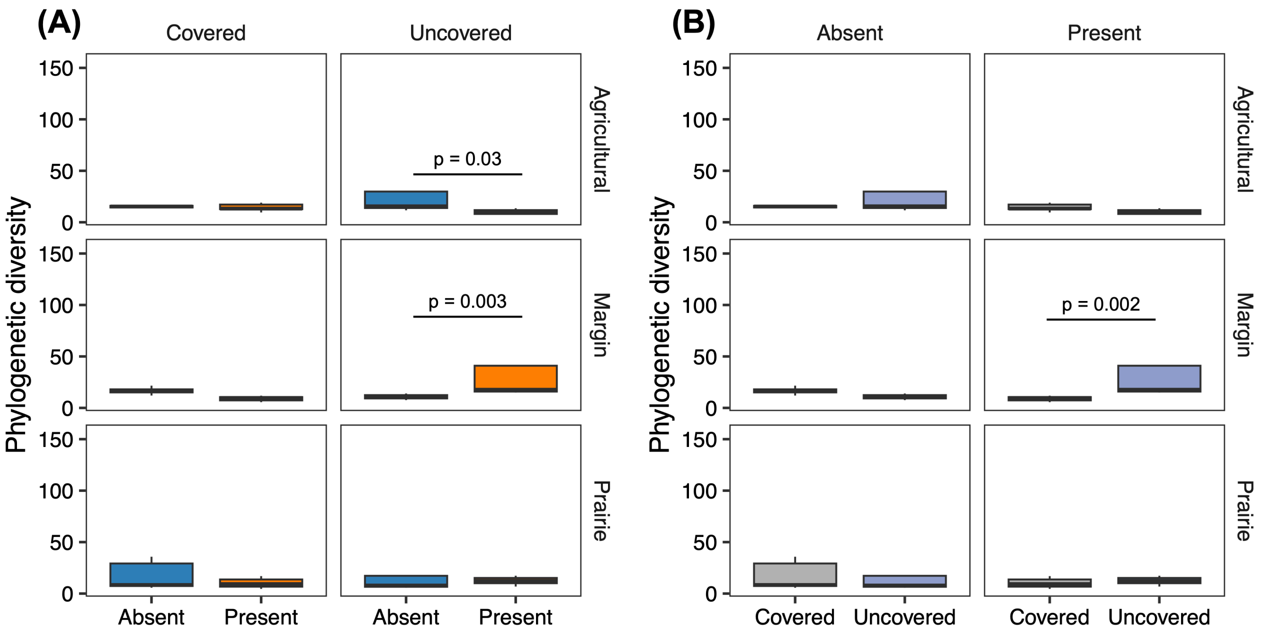

Figure S1. Phylogenetic diversity in samples in the rhizosphere compartment. **(A)** Comparison between samples exposed to herbivory (present) and control (absent) for each combination of soil inoculum (agricultural, margins, prairie) and coverage (covered, uncovered). **(B)** Comparison between samples from microcosms with covered and uncovered surface, for each combination of soil inoculum (agricultural, margins, prairie) and herbivory (present, absent). Post-hoc p-values are FDR-corrected.

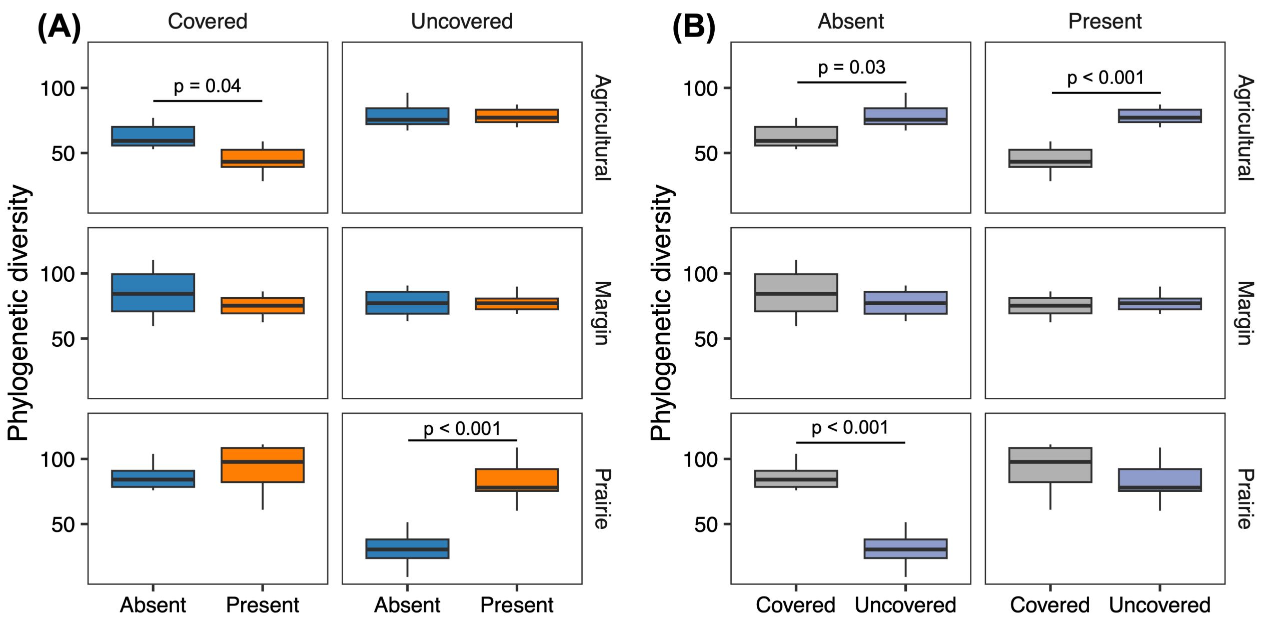

Figure S2. Phylogenetic diversity for samples in the <u>root compartment</u>. **(A)** Comparison between samples exposed to herbivory (present) and control (absent) for each combination of soil inoculum (agricultural, margins, prairie) and coverage (covered, uncovered). **(B)** Comparison between samples from microcosms with covered and uncovered surface, for each combination of soil inoculum (agricultural, margins, prairie) and herbivory (present, absent). Post-hoc p-values are FDR-corrected.

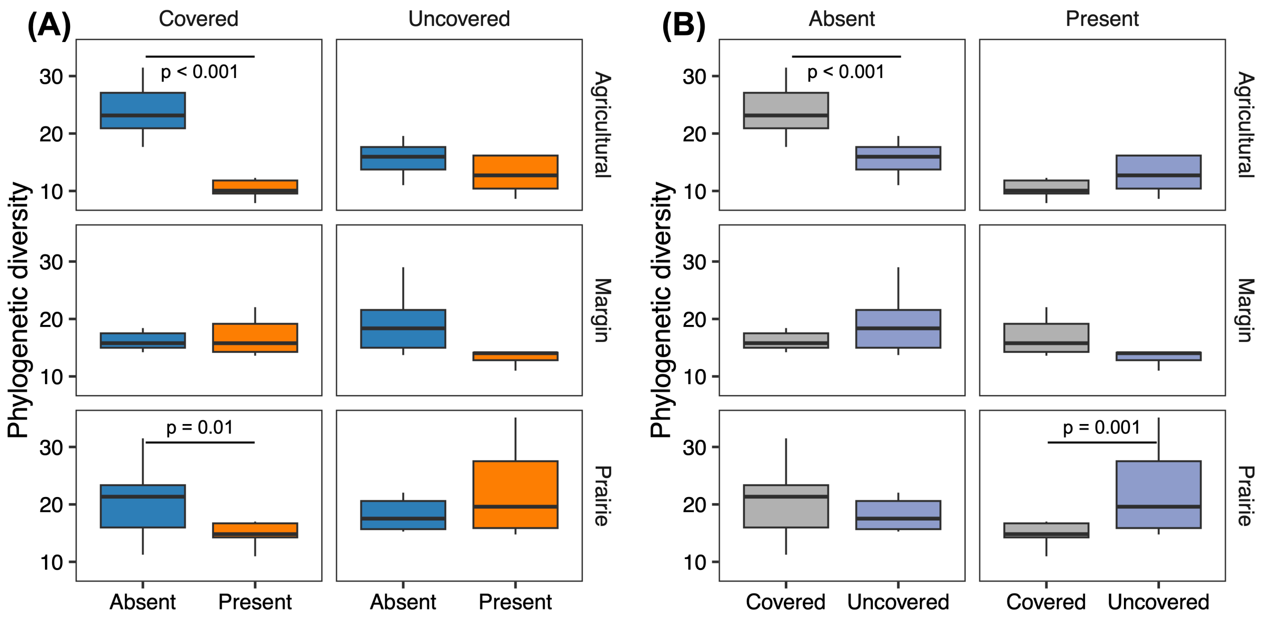

Figure S3. Phylogenetic diversity for samples in the <u>leaf compartment</u>. (A) Comparison between samples exposed to herbivory (present) and control (absent) for each combination of soil inoculum (agricultural, margins, prairie) and coverage (covered, uncovered). (B) Comparison between samples from microcosms with covered and uncovered surface, for each combination of soil inoculum (agricultural, margins, prairie) and herbivory (present, absent). Post-hoc p-values are FDR-corrected.

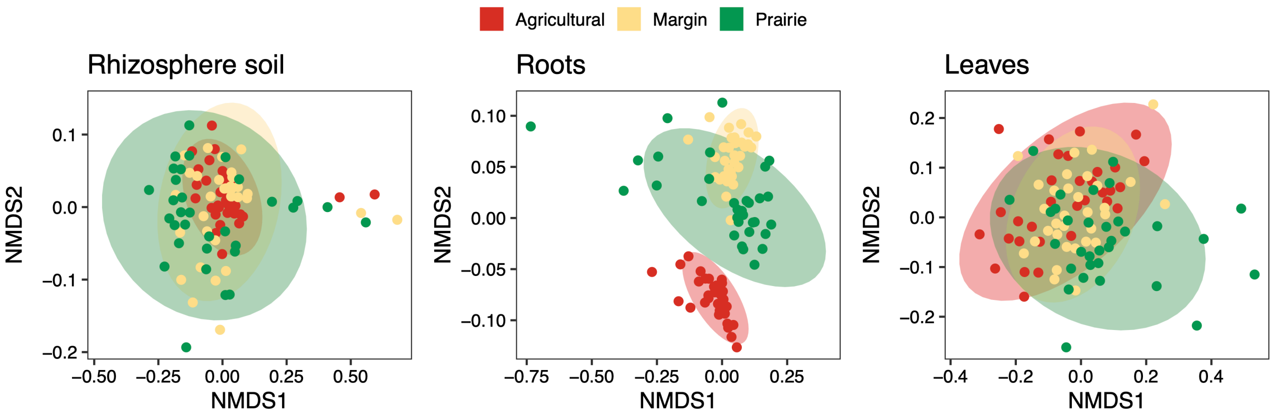

Figure S4. NMDS plots of bacterial community Unifrac distance matrix for each compartment. Points and 95% CI ellipses are coloured by soil inoculum.

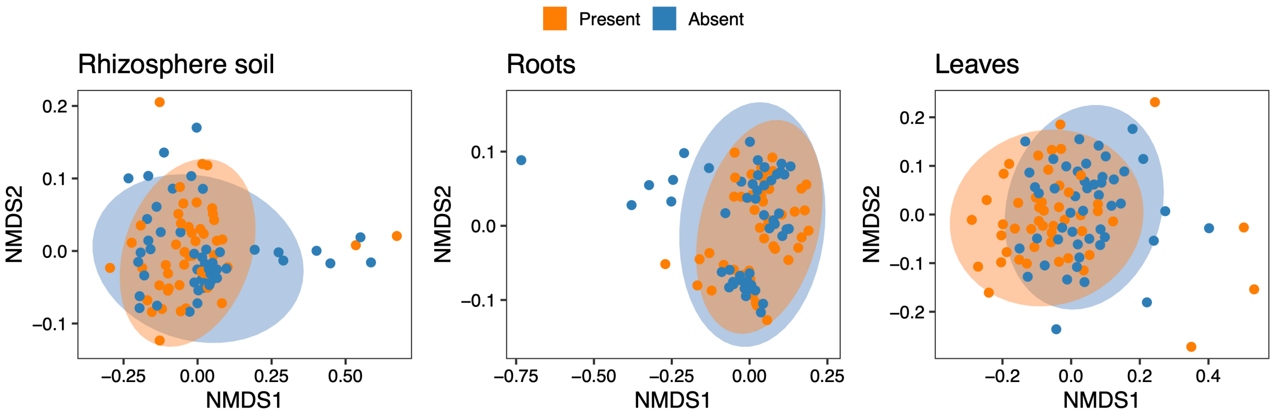

Figure S5. NMDS plots of bacterial community Unifrac distance matrix for each compartment. Points and 95% CI ellipses are coloured by presence or absence of herbivore.

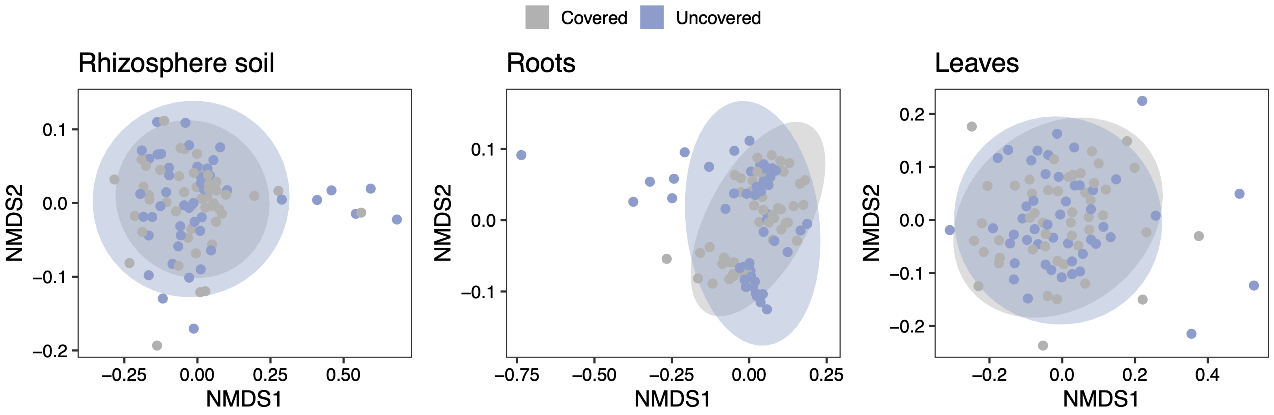

Figure S6. NMDS plots of bacterial community Unifrac distance matrix for each compartment. Points and 95% CI ellipses are coloured by presence or absence of soil cover.

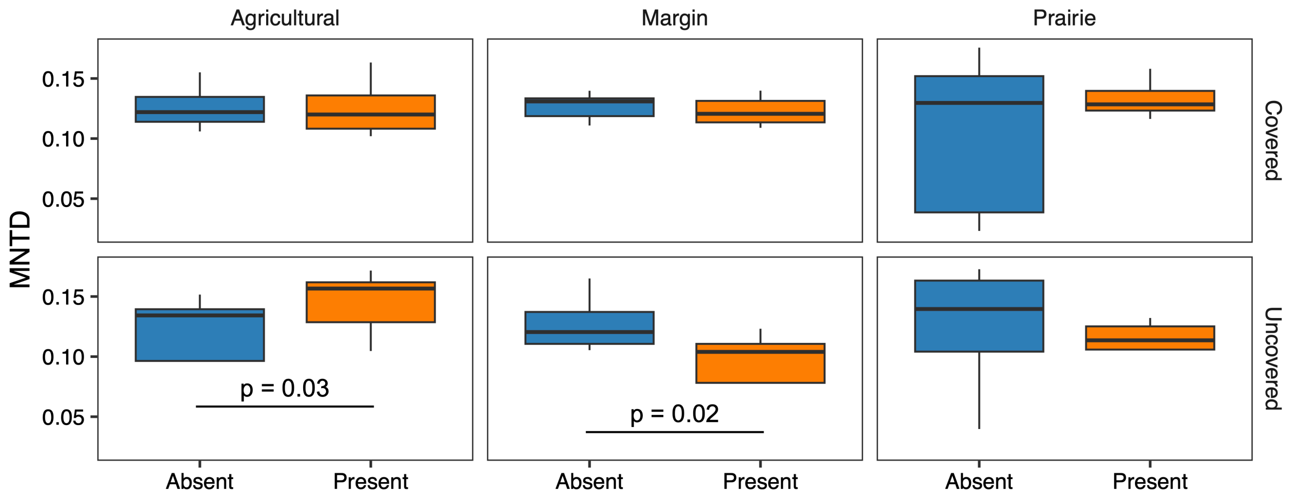

Figure S7. Mean Nearest Taxon Distance (MNTD) for samples in the rhizosphere compartment. Comparison between samples from microcosms with herbivores or control group, for each combination of soil inoculum (agricultural, margins, prairie) and cover treatment (covered, uncovered). Post-hoc p-values are FDR-corrected.

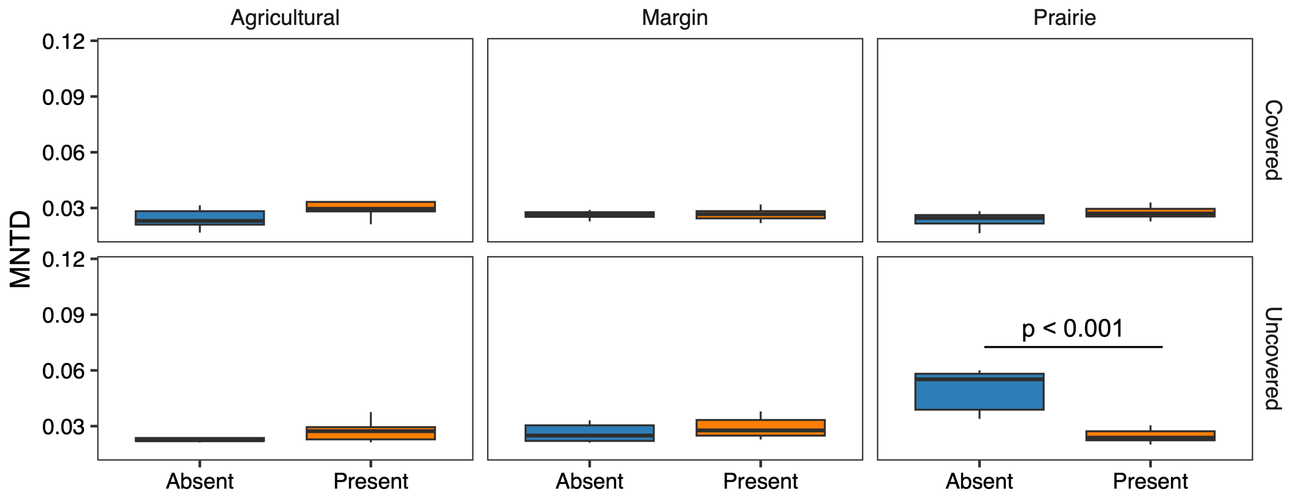

Figure S8. Mean Nearest Taxon Distance (MNTD) for samples in the root compartment. Comparison between samples from microcosms with herbivores or control group, for each combination of soil inoculum (agricultural, margins, prairie) and cover treatment (covered, uncovered). Post-hoc p-values are FDR-corrected.

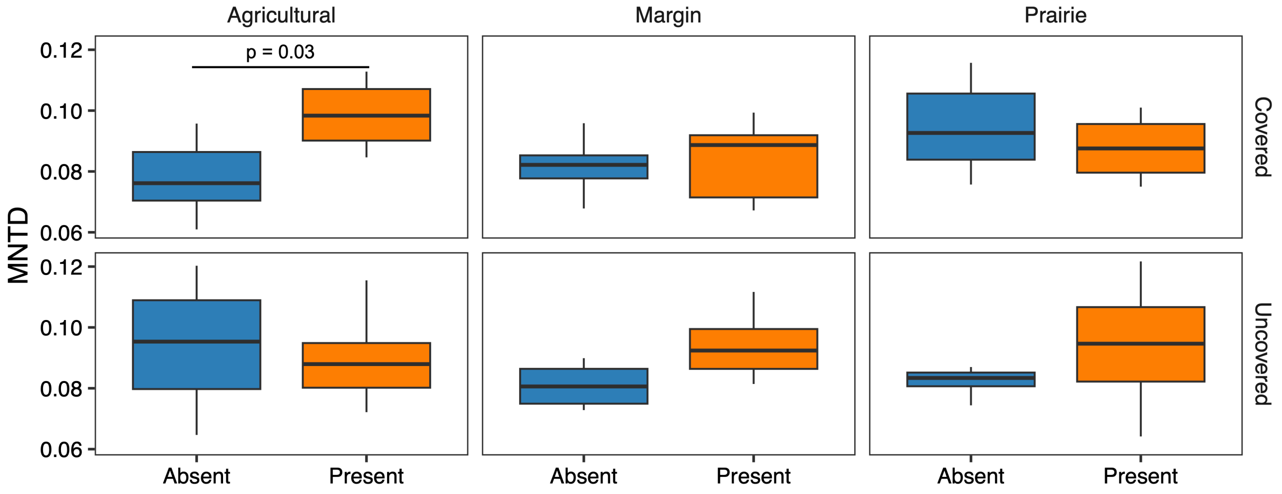

Figure S9. Mean Nearest Taxon Distance (MNTD) for samples in the leaf compartment. Comparison between samples from microcosms with herbivores or control group, for each combination of soil inoculum (agricultural, margins, prairie) and cover treatment (covered, uncovered). Post-hoc p-values are FDR-corrected.

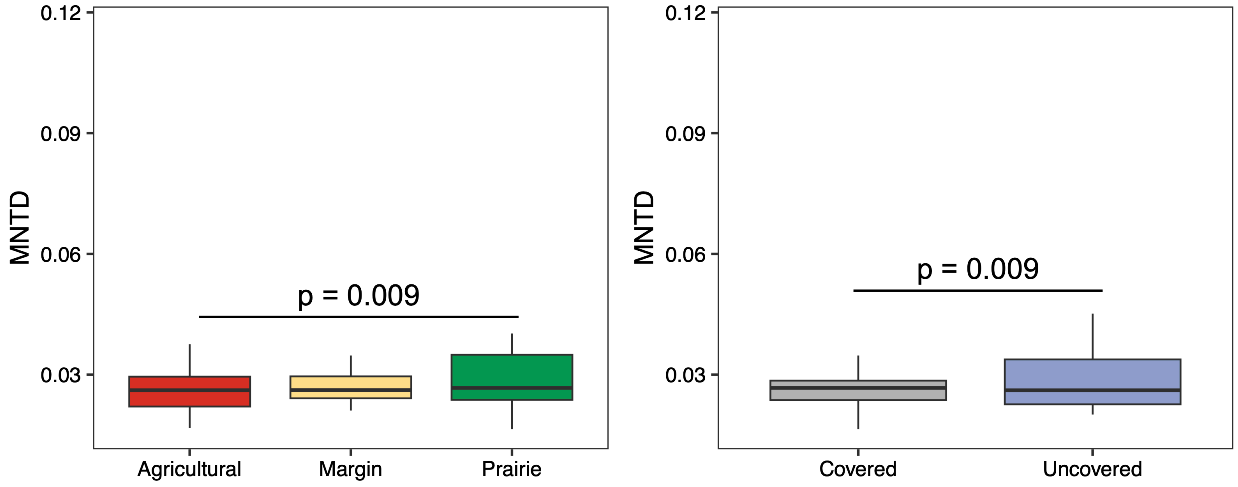

Figure S10. Mean Nearest Taxon Distance (MNTD) for samples in the root compartment. **(A)** Comparison between samples from microcosms with different soil inocula (agricultural, margins, prairie) and **(B)** cover treatment (covered, uncovered). Post-hoc p-values are FDR-corrected.

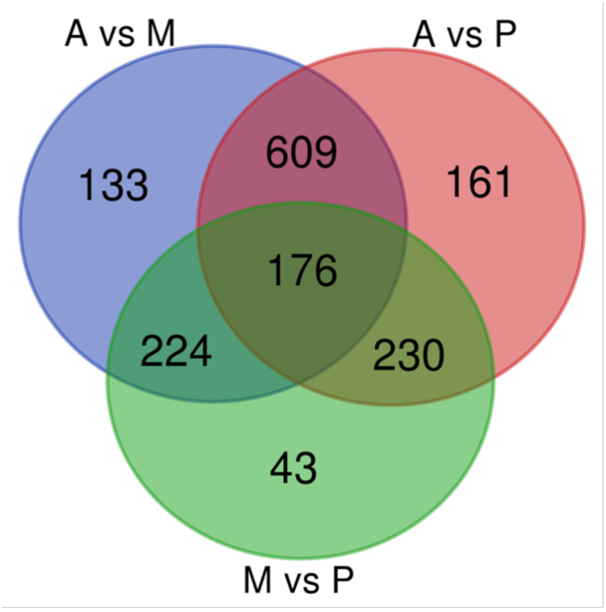

Figure S11. Venn diagram comparing ASVs which abundance was influenced by the soil inoculum across all pairwise comparisons: agricultural soil vs field margins (A vs M), agricultural soil vs prairie soil (A vs P), field margins vs prairie soil (M vs P).

Supplementary tables

Table S1. Results from a linear mixed-effects model for each compartment (rhizosphere soil, roots, leaves, herbivore) testing the effect of soil inoculum (S; agricultural, margins, prairie), herbivory (H; present, absent), coverage (T; present, absent), and all their interactions on the phylogenetic diversity index. The variance explained (R^2) is reported for each of the single factor.

		Rhizosphere			Roots			Leaves			Herbivore		
**Factor**	df	χ^2	p	R^2	χ^2	p	R^2	χ^2	p	R^2	χ^2	p	R^2
**Soil (S)**	2	0.51	0.77	0.005	8.4	0.01	0.038	9.17	0.01	0.056	7.31	0.02	0.149
**Herbivory (H)**	1	0.48	0.48	0.004	4.39	0.03	0.024	14.47	0.001	0.092			
**Coverage (C)**	1	2.13	0.14	0.019	1.25	0.26	0.005	0.03	0.84	0.0002	0.07	0.78	0.001
**S × H**	2	6.81	0.03		32.49	0.001		13.98	0.001				
**S × C**	2	2.5	0.28		56.26	0.001		6.4	0.04		0.89	0.63	
**H × C**	1	1.13	0.28		17.34	0.001		8.15	0.004				
**S × H × C**	2	8.46	0.01		6.18	0.04		16.58	0.001				

Table S2. PERMANOVA post-hoc pairwise comparison between covered and control groups for each herbivory x soil inoculum combination, for both rhizosphere soil and roots bacterial microbiota. Values represent FDR-corrected p-values.

		Rhizosphere soil	Roots	Leaves
**Herbivory**	*Agricultural*	0.026	0.001	0.012
	Margin	0.004	0.037	0.022
	Prairie	0.166	0.001	0.005
**Control**	*Agricultural*	0.005	0.001	0.005
	Margin	0.002	0.047	0.066
	Prairie	0.271	0.039	0.203

Table S3. PERMANOVA post-hoc pairwise comparison between herbivory and control groups for each coverage x soil inoculum combination, for both rhizosphere soil and roots bacterial microbiota. Values represent FDR-corrected p-values.

		Rhizosphere soil	Roots	Leaves
**Covered**	*Agricultural*	0.004	0.008	0.003
	Margin	0.002	0.009	0.012
	Prairie	0.045	0.029	0.003
**Control**	*Agricultural*	0.004	0.019	0.006
	Margin	0.002	0.804	0.127
	Prairie	0.046	0.001	0.1

Table S4. Results from a linear mixed-effects model for each compartment (rhizosphere soil, roots, leaves, herbivore) testing the effect of soil inoculum (S; agricultural, margins, prairie), herbivory (H; present, absent), coverage (T; present, absent), and all their interactions on the MNTD index.

		Rhizosphere		Roots		Leaves		Herbivore	
**Factors**	**df**	χ^2	**p**	χ^2	**p**	χ^2	**p**	χ^2	**p**
**Soil (S)**	2	1.15	0.56	10.85	0.004	2.8	0.24	1.98	0.37
**Herbivory (H)**	1	0.21	0.64	1.57	0.21	3.81	0.05		
**Coverage (C)**	1	0.42	0.51	7.07	0.007	0.19	0.66	0.16	0.68
**S × H**	2	6.01	0.04	17.81	0.001	0.9	0.63		
**S × C**	2	2.26	0.32	15.57	0.001	1.08	0.58	1.54	0.46
**H × C**	1	0.71	0.39	12.16	0.001	0.18	0.66		
**S × H × C**	2	6.64	0.03	18.95	0.001	7.93	0.01		

Table S5. Results from chi-squared tests comparing the number of ASVs unique to a compartment or shared between compartments in plants grown on pots with covered and uncovered soil surface. See Figure 3.

**Intersection**	**covered**	**uncovered**	χ^2	**p**
herbivore	83	101	1.76	0.185
herbivore-leaves	1	2	0.33	0.564
herbivore-leaves-rhizosphere	2	3	0.20	0.655
herbivore-leaves-roots	95	19	50.67	0.001
herbivore-leaves-roots-rhizosphere	250	333	11.82	0.001
herbivore-rhizosphere	2	9	4.45	0.035
herbivore-roots	220	110	36.67	0.001
herbivore-roots-rhizosphere	87	262	87.75	0.001
leaves	48	54	0.35	0.552
leaves-rhizosphere	7	23	8.53	0.003
leaves-roots	148	89	14.68	0.001
leaves-roots-rhizosphere	66	164	41.75	0.001
rhizosphere	64	460	299.26	0.001
roots	3914	2821	177.37	0.001
roots-rhizosphere	183	1013	576.01	0.001

Supplementary Data 1. ASVs in the root compartment significantly influenced by soil inoculum as pairwise contrasts between agricultural soil and field margins (sheet 1, A vs M), agricultural soil and prairie soil (sheet 2, A vs P), field margins and prairie (sheet 3, M vs P). Includes also ASVs in the root compartment significantly influenced by soil cover. **Table is attached as xlsx file**.
