# Supplementary material for: Soil microbiota and herbivory drive the assembly of tomato plant-associated microbial communities through different mechanisms
Source: Commun Biol. 2024 May 13;7:564. doi: 10.1038/s42003-024-06259-6 (PMC11091179; doi:10.1038/s42003-024-06259-6)
Supplement: Supplementary file 3 — Description of Supplementary Materials [file 42003_2024_6259_MOESM3_ESM.pdf]

Description of Additional Supplementary Files

File name: Supplementary Data 1

Description: ASVs in the root compartment significantly influenced by soil inoculum as pairwise contrasts between agricultural soil and field margins (sheet 1, A vs M), agricultural soil and prairie soil (sheet 2, A vs P), field margins and prairie (sheet 3, M vs P). Includes also ASVs in the root compartment significantly influenced by soil cover.
